# Human cytomegaloviral multifunctional protein kinase pUL97 impairs zebrafish embryonic development and increases mortality

**DOI:** 10.1038/s41598-019-43649-x

**Published:** 2019-05-10

**Authors:** Salvador Cazorla-Vázquez, Mirjam Steingruber, Manfred Marschall, Felix B. Engel

**Affiliations:** 1 Experimental Renal and Cardiovascular Research, Department of Nephropathology, Institute of Pathology, Friedrich-Alexander-Universität Erlangen-Nürnberg (FAU), Schwabachanlage 12, 91052 Erlangen, Germany; 2 Institute for Clinical and Molecular Virology, Friedrich-Alexander-Universität Erlangen-Nürnberg (FAU), Schlossgarten 4, 91054 Erlangen, Germany

**Keywords:** Disease model, Viral infection

## Abstract

Cytomegalovirus is a worldwide-distributed human pathogen, which is the leading cause of congenital virus infection, affecting 0.5 to 2% of live births. To date, it is largely unclear which molecular mechanisms underlie the symptomatic outcomes. This is mainly due to species specificity and limited homology among cytomegalovirus genomes. As it is not possible to infect model organisms with human cytomegalovirus, the aim of this study was to develop a heterologous system allowing in the future the elucidation of the pathological role of individual viral proteins. As a model organism the zebrafish has been chosen due to its ease of manipulation and characterization as well as its large offspring. As cytomegalovirus model protein, pUL97 was characterized because it is multiply involved in virus-host interaction. Here, we show in zebrafish embryos, that (i) pUL97 can be expressed in zebrafish, (ii) increasing pUL97 expression levels quantitatively correlate with both minor and major pathological defects, (iii) pUL97 expression impairs cell cycle progression and induces cell death, (iv) active pUL97, but not an inactive mutant, induces excess mortality, and (v) co-administration of a pUL97 inhibitor reduces embryonic pathology. Collectively, these data indicate the suitability of zebrafish to elucidate the pathological role of human cytomegaloviral proteins.

## Introduction

Human cytomegalovirus (HCMV, family *Herpesviridae*) represents a major, worldwide-distributed human pathogen. Primary HCMV infection of the immunocompetent host frequently remains asymptomatic, but severe disease can occur upon infection of immunonaive and immunocompromised individuals, such as neonates, transplant recipients and cancer or AIDS patients^1^. Importantly, HCMV is the leading cause of congenital virus infection (‘congenital CMV’), affecting 0.5 to 2% of live births, a fact that has frequently been underestimated in the field of broader scientific and public perception^1–3^. The recent Zika virus outbreak in Brazil fortunately significantly increased the awareness of the potential of intrauterine viral infections to lead to severe pathological defects^4^. The incidence of HCMV transmission to the fetus depends on maternal serostatus, with transmission rates of ∼30–40% in primary and ∼1.7% in recurrent infections^3^. Approximately 10% of infected newborns are symptomatic at birth, and up to 15% of those asymptomatic at birth develop delayed HCMV-related disease manifestations in their early years. Congenital CMV is associated with a wide range of neurodevelopmental disabilities, including hearing and vision loss and mental retardation, as well as structural brain abnormalities including microcephaly, hydrocephalus, ventriculomegaly, and periventricular cerebral calcifications^5,6^.

HCMV pathogenicity is often directly linked with the efficiency of viral replication and viral load^2^. Moreover, an interference of HCMV with cellular signaling pathways and immune cell activation has been discussed on various levels^3,7–9^. However, the role of individual HCMV-encoded proteins in HCMV pathogenicity is still poorly understood; in particular for congenital CMV. This is mainly because HCMV cannot be studied in experimental animals as CMVs are highly species-specific and the study of human tissues *ex vivo* is difficult. Moreover, each CMV species is adapted to its respective host species, which is reflected by the limited homology of the viral genomes of CMVs infecting small animal models (e.g. mouse, rat or guinea pig). In addition, it has been shown that the models utilized to study congenital CMV exhibit disadvantages such as limited CMV transmission to the fetus (mouse, rat), lack of genetic models (guinea pig), low numbers of offspring and/or *in utero* development^10,11^. Therefore, it is important to develop new strategies, which allow the characterization of the congenital pathological potential and disease mechanism of cytomegaloviral proteins.

In contrast to the currently used CMV models, the zebrafish offers several advantages in the context of congenital CMV research such as large offspring (200 to 300 eggs per female and week) with an external and rapid development (precursors to all major organs appear within 36 hours post-fertilization, hpf), optical clarity during embryogenesis, low maintenance costs, usability for large-scale forward genetics and chemical screens, availability of large numbers of mutant and reporter lines, a fully sequenced genome, and ease of genetic manipulation^12,13^. In addition, it has previously been reported that zebrafish is a suitable model to study human pathophysiology^13–18^, including the immune response to human viral infections^19^. Examples are immune responses to influenza A virus infection^20,21^, neuroinvasion by the alphaviruses chikungunya virus and Sindbis virus^22^, studies on hepatitis C virus replication^23^, herpes simplex virus 1 entry^24^, as well as intrahepatic cholangiocarcinoma associated with hepatitis B and C virus^25^. Also, the regulatory potency of protein kinases, including those involved in herpesviral replication^7^, has been addressed in the zebrafish model recently^26^. However, also zebrafish cannot be infected with HCMV. Therefore, the aim of this study was to determine whether HCMV-encoded proteins can be expressed as functional proteins in zebrafish allowing in the future the elucidation of the pathological role of individual human cytomegaloviral proteins.

The HCMV serine/threonine protein kinase pUL97 has been shown to be multiply involved in virus-host interaction^27^. For example, pUL97 is associated with the core nuclear egress complex and phosphorylates core members^28,29^. Further, pUL97 phosphorylates the most abundant tegument protein of HCMV virions, the phosphoprotein pp65, which mediates the upload of other virion constituents and contributes to particle integrity^30^. Finally, pUL97 is a viral cyclin-dependent kinase ortholog that interacts with host proteins involved in cell cycle regulation, including cyclins^31–33^, tumor suppressor Rb^34,35^, nuclear lamins A/C^36,37^, and histones^38^. As pUL97 does not only regulate HCMV replication, but influences also host cellular functions we utilized pUL97 as a model HCMV protein.

In the present report, we show that plasmid-driven ectopic expression of the human cytomegaloviral multifunctional protein kinase pUL97 in zebrafish zygotes resulted in mosaic patterns of expression in embryonic tissues. The induction of light and severe abnormalities in development could be measured in qualitative and quantitative terms. In addition, embryonic pUL97 expression was associated with impaired cell cycle progression, increased cell death and excess mortality in the zebrafish model. These effects were markedly reduced upon ectopic expression of a catalytically inactive mutant of pUL97 as well as by coadministration of a pharmacological pUL97 inhibitor. Collectively, our data indicate that the zebrafish model is suitable for the analysis of human herpesviral proteins during embryonic development.

## Results

### pUL97 can be expressed in zebrafish

**Figure 1:**
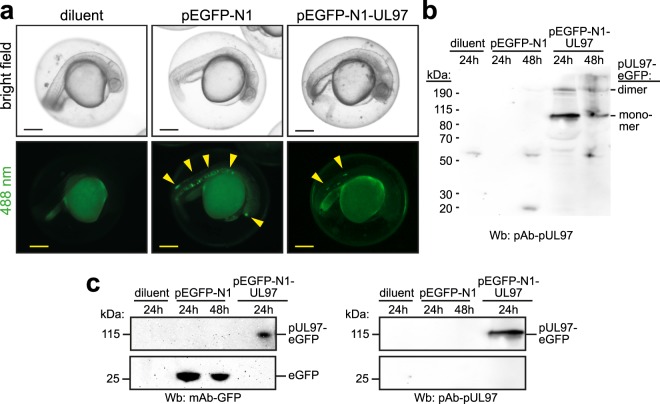
Mosaic expression of pUL97 in zebrafish embryos. Single cell-staged embryos were injected with nuclease-free water (diluent) or plasmids (50 ng/µl) encoding eGFP and pUL97-eGFP. (**a**) Microscopic analysis at 24 hpf indicating mosaic expression of eGFP or pUL97-eGFP (yellow arrowheads). (**b**,**c**) Western blot analysis using total lysates of zebrafish embryos (~6 embryos per lane) probed with anti-pUL97 and anti-GFP antibodies. Arrowheads: ectopic expression. Scale bars: 250 µm.

HCMV is a human-specific pathogen and thus cannot be utilized to directly study in model systems the molecular mechanisms underlying HCMV-induced pathologies. Here, we addressed the hypothesis that zebrafish is an appropriate system to investigate the role of individual HCMV proteins in HCMV-related pathologies. As the zebrafish is an established model system for embryology^12^ and HCMV is the leading cause of congenital virus infection^1–3^, we tested first whether the HCMV kinase pUL97 can be expressed in zebrafish. For this purpose, single cell-staged embryos were injected with nuclease-free water (diluent) or plasmids (50 ng/µl) encoding eGFP or pUL97-eGFP. Microscopic analysis at 24 hpf revealed mosaic GFP expression for both plasmid injections suggesting that pUL97 might be expressed in zebrafish embryos (Fig. 1a). To confirm the presence of full-length pUL97, total protein lysates were subjected to western blot analysis (~6 embryos/lane) using anti-pUL97 antibodies (Fig. 1b). This analysis revealed protein bands corresponding to pUL97-eGFP monomers and dimers (note, the formation of pUL97 dimers has already previously been reported^39^ and dimers are occasionally seen for pUL97^39–41^ as well as other viral proteins, probably due to insufficient denaturing and reducing conditions^42,43^). Subsequently, we determined the levels of transgene expression from pEGFP-N1 and pEGFP-N1-UL97 transfections by western blot analysis utilizing anti-pUL97 and anti-GFP antibodies (Fig. 1c and Supplementary Fig. 1). Our data indicate that the protein expression level of pUL97-eGFP is lower than of GFP, which is possibly due to the fact that 50 ng/µl pEGFP-N1-UL97 (6.8 kb) equals a lower plasmid copy number than 50 ng/µl of pEGFP-N1 (4.7 kb). Other possible explanations are differences in RNA or protein stability as well as translation efficiency. Collectively, our data demonstrate that pUL97 can be expressed in zebrafish embryos.

### pUL97 impairs zebrafish embryonic development and increases mortality

**Figure 2:**
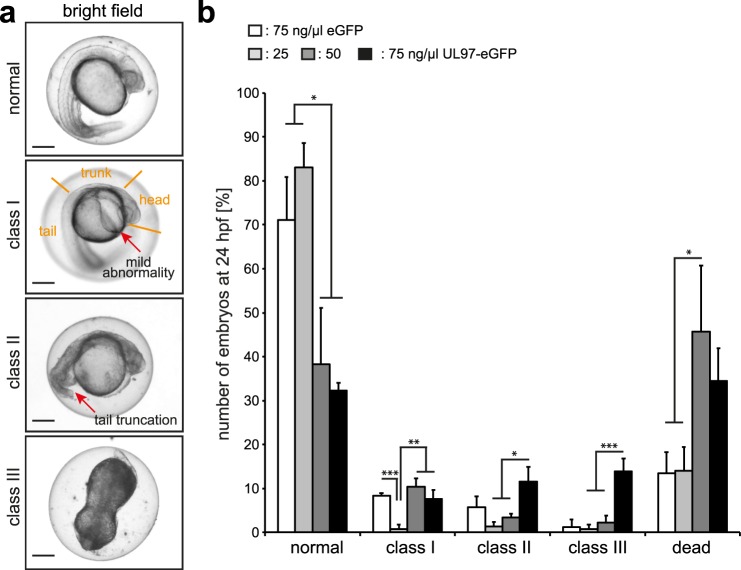
Ectopic mosaic expression of pUL97 induces developmental defects and mortality in zebrafish embryos. Single cell-staged embryos were injected with plasmids encoding eGFP or pUL97-eGFP at increasing concentrations as indicated. At 24 hpf, embryos were categorized in phenotype classes and quantitated. (**a**) Bright field images of the three phenotype classes. (**b**) Quantitative analysis. For each condition and independent experiment 46 to 181 embryos were analyzed (in total 1071 embryos in three independent experiments). Data are mean ± SD. Scale bars: 250 µm. *p < 0.05, **p < 0.02, ***p < 0.01.

To address the question whether ectopic expression of pUL97 in zebrafish results in developmental defects, single cell-staged zebrafish embryos were injected with varying concentrations of plasmids (mosaic expression, Fig. 2) or RNA (global expression, Supplementary Fig. 2) encoding eGFP or pUL97-eGFP as indicated. While previously it has been indicated that pUL97 kinase activity is robust towards N- and C-terminal fusions^41^, we confirmed by *in vitro* kinase assays that the here utilized tagged pUL97 versions exhibit normal kinase activity (Supplementary Fig. 3). Phenotypic characterization of zebrafish embryos was performed at 24 hpf based on bright field images. The following phenotype classes were defined and quantitated: (i) normal, no obvious phenotype; (ii) class I, preserved tail, trunk, head structure exhibiting light abnormalities such as edemas, irregular shapes; (iii) class II, tail truncation; (iv) class III, severe abnormalities; (v) dead embryos (Fig. 2a). Quantitative analysis of these phenotypes revealed that ectopic mosaic expression of pUL97-eGFP at intermediate and high plasmid concentrations markedly increased mortality (Fig. 2b). In regards to developmental defects, it was apparent that already high concentrations of the control plasmid (75 ng/µl pEGFP-N1) *per se* caused mild class I phenotypes, but almost no severe class III phenotypes. Low concentrations of the pUL97-encoding plasmid (25 ng/µl pEGFP-N1-UL97) did only exert minor effects on zebrafish development. Yet, with increasing concentrations of pUL97 ectopic expression, the percentage of zebrafish embryos exhibiting more severe phenotypes (class II and class III) or death increased significantly (Fig. 2b). Global expression due to RNA injection (200 pg) in one cell-staged embryos resulted at 24 hpf in a more severe phenotype than in mosaic expression. At 24 hpf, more than 90% of the pUL97-eGFP-expressing embryos lost all morphological characteristics and the mortality raised to >55% (Supplementary Fig. 2a). Note, that eGFP was expressed in a ubiquitous, smooth pattern while UL97-eGFP was expressed in a ubiquitous, dotty pattern (Supplementary Fig. 2a). This might be because pUL97 contains a NLS sequence mediating nuclear translocation^44^.

**Figure 3:**
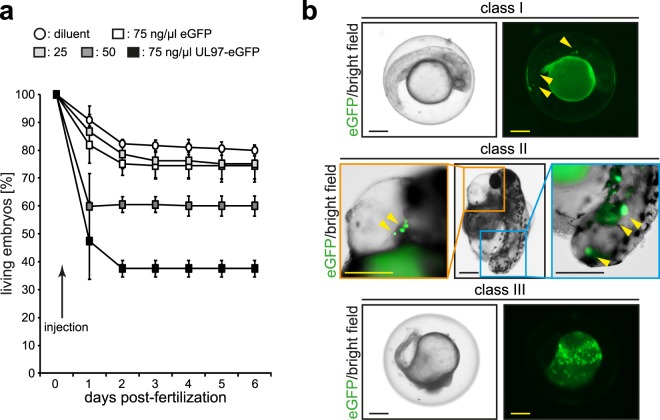
Local pUL97 expression is associated with pathological phenotypes. (**a**) Quantitative analysis of zebrafish survival upon expression of eGFP or pUL97-eGFP at indicated concentrations until 6 dpf. Note, the data from 1 day post-fertilization for injections of 50 and 75 ng/µl UL97-eGFP was significantly different (p < 0.05) from the 2 control groups as well as injections with 25 ng/µl UL97-eGFP. (**b**) Microscopic images visualizing an association of pUL97-eGFP expression with developmental defects. Data are mean ± SD. Scale bars: 250 µm.

A time-resolved analysis of embryo survival upon expression of pUL97-eGFP revealed that maximum mortality was reached at 48 hpf (Fig. 3a). These data confirmed our finding that increasing pUL97-eGFP is correlated with increased mortality. In some cases, the expression of pUL97-eGFP was associated with an affected organ, such as cardiac edema or tail truncation (Fig. 3b). Moreover, class III embryos were characterized by a large number of pUL97-eGFP-positive cells. Yet, in many cases the affected organs did not exhibit pUL97-eGFP-positive cells.

### pUL97 expression impairs cell cycle progression and increases cell death

**Figure 4:**
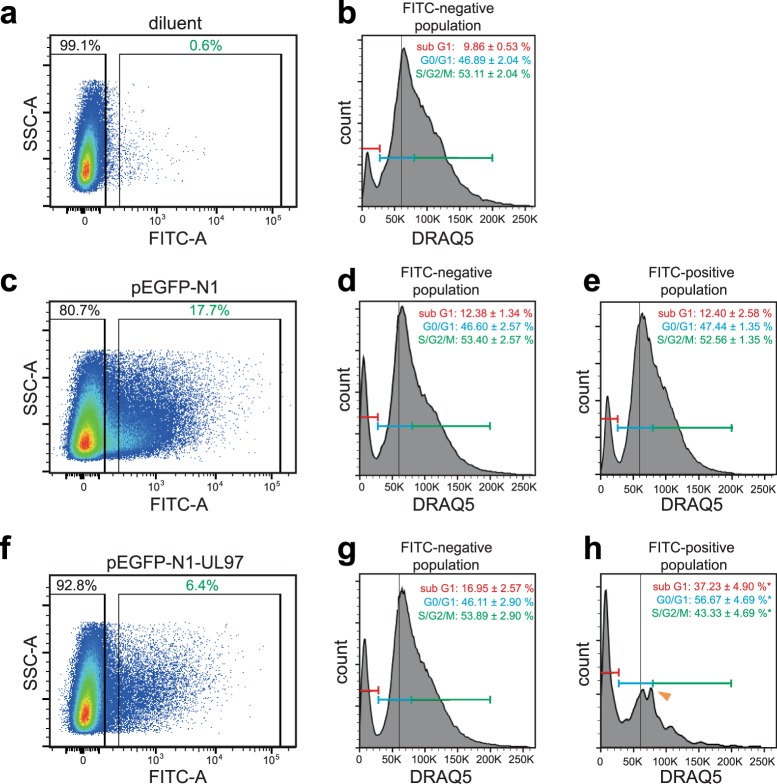
pUL97-eGFP expression impairs cell cycle progression and increases cell death. Representative FACS analysis (n = 3). Single cell-staged embryos were injected with nuclease-free water (diluent) or plasmids (75 ng/µl) encoding for eGFP or pUL97-eGFP, were dissociated at 24 hpf into living single cell suspensions, stained with DRAQ5, and analyzed by FACS. (**a**,**c**,**f**) Side scatter plots, which were used to gate cells according to their eGFP fluorescence intensity (FITC-A). (**b**,**d**,**g**) Cell cycle profile of eGFP-negative populations. (**e**,**h**) Cell cycle profile of eGFP-positive populations. Note, the percentage of the sub G1 cells is related to all measured cells, while the given percentages in the different cell cycle phase is related to all cells except the sub G1 population. Arrowhead: indicates an additional post G1 peak, which was present in all cell cycle profiles of pUL97-eGFP expressing cells. For each condition and independent experiment, 20 to 32 embryos were pooled and analyzed (in total 226 embryos in three independent experiments). Data are mean ± SD. *p < 0.05.

pUL97 has been postulated to play a major regulatory role in HCMV-induced pseudomitosis, a term used to refer to an early S phase arrest in HCMV-infected cells that offers favorable conditions for viral replication^33,45^. Currently, it is hypothesized that pUL97 interferes with the G1/S phase checkpoint transition by phosphorylation and inactivation of tumor suppressor Rb^34,35^ and/or modulating host cyclin-dependent kinase function^33^. In order to assess whether pUL97 expression affects cell cycle progression during zebrafish development, plasmid-injected zebrafish embryos were harvested at 24 hpf, disintegrated into living single cell suspensions, and analyzed by fluorescent-activated cell sorting (FACS) upon DNA staining with the live DNA staining dye DRAQ5 (Fig. 4). This analysis identified a clear G0/G1 phase peak. Yet, in contrast to FACS analyses of fixed cell lines, embryonic zebrafish cells in S phase and G2 phase did not appear as additional peaks as previously reported^46^. eGFP-negative cells in all groups (diluent, pEGFP-N1, pEGFP-N1-UL97) as well as eGFP-positive cells in the pEGFP-N1-group exhibit a similar cell cycle profile (G0/G1: ~47%, S/G2/M: ~53%, Fig. 4b,d,e,g). In contrast, pUL97-eGFP expressing cells exhibited an altered cell cycle profile with an increased G0/G1 population (56.67 ± 4.69%, Fig. 4h). Furthermore, in all FACS profiles of pUL97-eGFP expressing cells appeared a second peak shortly after the G0/G1 peak (arrowhead, Fig. 4h). Collectively, these data suggest that pUL97-eGFP expression in zebrafish cells impairs cell cycle progression by delaying G1/S transition.

In addition to the altered cell cycle profile, our FACS analyses indicated that pUL97-eGFP expression causes increased cell death. DRAQ5 staining is reduced in dead/dying cells due to compromised DNA resulting in a so-called sub G1 peak. While eGFP expression increased the sub G1 population only slightly in comparison to eGFP negative cells in diluent and pEGFP-N1-injected embryos (12.40 ± 2.58% vs. 9.86 ± 0.53% and 12.38 ± 1.34%, respectively), pUL97-eGFP expression significantly increased the sub G1 population (37.23 ± 4.90%) (Fig. 4). Notably, also eGFP-negative cells in pEGFP-N1-UL97-injected embryos showed an increased sub G1 peak (16.95 ± 2.57%), which might be a secondary response of cells neighboring dying cells (Fig. 4g).

### The pUL97-inflicted developmental defects and mortality are kinase-dependent

**Figure 5:**
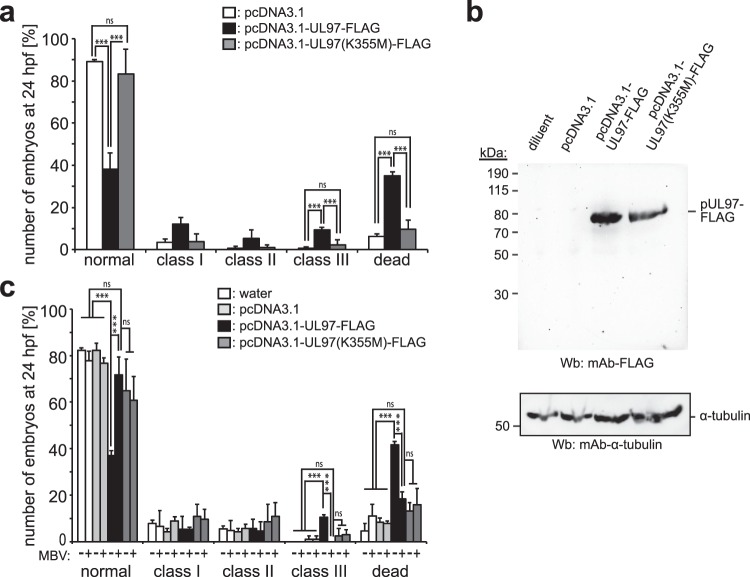
pUL97-inflicted developmental defects and mortality are kinase-dependent. (**a**) Single cell-staged embryos were injected with plasmids encoding active pUL97-FLAG, the inactive mutant pUL97(K335M)-FLAG or empty vector (75 ng/µl). At 24 hpf, embryos were categorized in phenotype classes as described in Fig. 2a and quantitated. (**b**) Western blot analysis using total lysates of 24 hpf zebrafish embryos (~6 embryos per lane) injected with diluent or plasmids as indicated. Blots were probed with anti-FLAG and anti-α-tubulin antibodies. (**c**) Single cell-staged embryos were injected with the indicated expression plasmids or empty vector pcDNA3.1 (75 ng/µl). Subsequently embryos were raised until 24 hpf in the presence or absence of 1 µM MBV and phenotypes were quantitated. For each condition and independent experiment 55 to 151 (**a**) and 19 to 36 (**b**) embryos were analyzed (in total 851 (**a**) or 673 (**b**) embryos in 3 independent experiments). Data are mean ± SD. ***p < 0.01.

In order to determine whether the detrimental effect of pUL97 on embryonic zebrafish development depends on its kinase activity, we utilized a catalytically inactive mutant (K355M^41^) as well as the pUL97 kinase inhibitor maribavir (MBV)^47,48^ (Fig. 5). As shown above for ectopic pUL97-eGFP expression (Fig. 2b), also the ectopic mosaic expression of pUL97-FLAG in zebrafish embryos induced developmental defects and increased mortality (Fig. 5a). In contrast, injection of plasmids encoding the pUL97 kinase-inactive mutant K355M-FLAG resulted in a markedly higher number of normal embryos as well as a lower number of embryos exhibiting class I to class III phenotypes and a lower mortality rate, at a similar level as upon injection of the plasmid backbone (pcDNA3.1). Similarly, also injection of RNA encoding the pUL97 inactive mutant pUL97(K355M)-eGFP did not significantly affect zebrafish development (Supplementary Fig. 2).

These data indicate that the observed effects of pUL97 are dependent on its kinase activity. Yet, the expression level of the kinase-dead mutant was slightly lower than of the wild type kinase (Fig. 5b). Thus, to confirm this conclusion, the effect of co-administration of the pharmacological pUL97 inhibitor MBV was analyzed (Fig. 5c). Quantitative evaluations demonstrated that MBV treatment of pUL97-expressing zebrafish embryos abolished the detrimental effects at a concentration of 1 µM (Fig. 5c).

## Discussion

This study was designed to determine whether the zebrafish model is a suitable system to study in the future the patho-regulatory function of HCMV-encoded proteins. Our data indicate, based on pUL97, that HCMV-encoded proteins can be expressed in zebrafish and may exhibit their pathological potential.

Our data demonstrate that the observed pUL97-induced phenotypes are dependent on its kinase function. As pUL97 is a viral cyclin-dependent kinase ortholog that interacts with host proteins involved in cell cycle regulation, including cyclins^31–33^, tumor suppressor Rb^34,35^, nuclear lamins A/C^36,37^, and histones^38^, we hypothesize that ectopic expression of pUL97 might interfere with cellular proliferation and possibly with other regulatory and metabolic host cell processes. Our FACS data suggest that pUL97 expression also interferes with cellular proliferation in the zebrafish model by delaying the G1/S transition. Unfortunately, it is not possible to check individual candidate pUL97 kinase targets in zebrafish as the zebrafish field suffers from a paucity of validated antibody reagents (usually reporter lines are utilized^49^). In future studies, it will thus be important to determine pUL97 kinase targets in zebrafish in order to determine if ectopic pUL97 expression results in a similar phosphoproteome as in human cells. For example, the SILAC approach could be utilized to determine the phosphoproteome directly^50^ and/or transgenic lines could be generated to identify pUL97 interaction partners by utilizing the BirA ligation system^51^.

Independent of the question whether the observed pUL97-induced developmental defects mirror human pathology, our system provides an easy read out system to test novel drugs aiming at the inhibition of pUL97 in an *in vivo* context. The advantages of zebrafish are the large off spring of 100dreds of embryos per breeding, the possibility of automated high throughput image analysis of zebrafish embryos^52^, and the fact that such assays are not considered to be animal experiments (until 6 days post-fertilization).

Besides testing the effect of ectopic expression of other HCMV-encoded proteins, it will be important to generate transgenic zebrafish lines that allow cell-type-specific as well as inducible expression of HCMV-encoded proteins utilizing for example tissue-specific promoters or heat shock promoter. This would not only allow to determine cell-type specific functions of cytomegaloviral proteins but also to express them at later stages during development to avoid early lethality. Moreover, such transgenic lines could be utilized for determining the effect on adult physiology by utilizing established functional assays for example to assess defects in vision^53^, hearing^54^, and behavior^55^. Given that only a small fraction of infected humans exhibit a phenotype, which can also differ significantly in terms of the affected organ, the zebrafish appears to be of advantage considering its large offspring.

Finally, we are aware that our system does not allow directly studying infection, amplification and release of CMV. Yet, our system has the potential to systematically elucidate the function of individual cytomegaloviral proteins or combinations of them in regards to their utilization of the cellular machinery of the host in an *in vivo* context. This will contribute to a better understanding of HCMV-associated pathologies and indirectly also provide candidate interactors and targets that are required for example for viral amplification and release.

## Methods

### Zebrafish

All methods were carried out in accordance with the guidelines from Directive 2010/63/EU of the European Parliament on the protection of animals used for scientific purposes.

### Zebrafish maintenance and breeding

Stocks of wild-type AB zebrafish were maintained and bred at standard laboratory conditions at 28.5 °C as described^56^. Zebrafish embryos were maintained at 28.5 °C in E3 medium (5 mM NaCl, 0.17 mM KCl, 0.33 mM CaCl_2_, 0.33 mM MgSO_4_, and 0.6 μM methylene blue).

### Microinjection and drug treatment

Single cell–stage embryos were injected into yolk (0.5 nl; PV820 Injector; World Precision Instruments) with the following plasmids: (i) pEGFP-N1 (Clontech Laboratories); (ii) pEGFP-N1-UL97 (ORF-UL97 cloned into vector pEGFP-N1/Clontech by using restriction sites sites *Eco*RI and *Sal*I; catalytic activity was verified by *in vitro* kinase assays); (iii) pcDNA3.1 (Invitrogen; pcDNA3.1); (iv) pcDNA3.1-UL97-FLAG and (v) pcDNA3.1-UL97(K355M)-FLAG^41^. In order to perform RNA injection experiments, the UL97-EGFP sequence from pEGFP-N1-UL97 was subcloned into pCS2+ by cold-fusion (insert: forward primer: 5′-TGCAGGATCCCATCGATTCGAATTCATGTCCTCCGCACTTCGGTCTCGGG-3′; reverse primer: 5′-TATAGTTCTAGAGGCTCGAGCTTGTACAGCTCGTCCATGCCG-3′; vector: forward primer: 5′-GCATGGACGAGCTGTACAAGCTCGAGCCTCTAGAACTATAGTGAG-3′, reverse primer: 5′-GTGCGGAGGACATGAATTCGAATCGATGGGATCCTGCAAAAAG-3′. The generated plasmid called pCS2+ UL97-EGFP was then used to generate a pCS2+ based plasmid to produce RNA encoding pUL97(K355M)-eGFP. For this purpose cold-fusion was performed with the forward primer 5′-CGCTATCGCGTGGTCATGGTGGCGCGTAAGCAC-3′ and reverse primer 5′- GTGCTTACGCGCCACCATGACCACGCGATAGCG-3′. RNA was obtained by *in vitro* transcription using mMESSAGE mMACHINE SP6 Transcription Kit (ThermoFisher, USA). Control injection was with the diluent, nuclease-free water. In order to inhibit pUL97 catalytic activity, embryos were cultivated after injection in E3 medium containing 1 µM of MBV.

### Microscopic analysis of zebrafish embryos

For bright-field and fluorescence microscopic analysis, images were captured using an EVOS fluorescence microscope (EVOS; AMG, USA). Embryos older than 24 hpf were anesthetized with 0.016% tricane methanesulfonate (Sigma-Aldrich, USA) in E3 medium.

### Fluorescent-activated cell sorting analysis

Zebrafish embryos at 24 hpf were dissociated into living cell suspensions as previously described^57^ and stained with 20 µM DRAQ5 (Abcam, UK) in PBS for 30 min. Subsequently, the cells were analyzed with a BD LSRFortessa (Becton Dickinson, USA) and analyzed with FlowJo software (Tree Star, USA). eGFP and DRAQ5 expression were detected with FITC and APC channels, respectively.

### Western blot analysis

Zebrafish embryos were collected 24 and 48 hours post-microinjection and dechorionated with 1% pronase (protease from *Streptomyces griseus*; Sigma-Aldrich, USA) in E3 medium at 37 °C in agarose-coated Petri dishes. Embryos were deyolked (55 mM NaCl, 1.8 mM KCl, 1.25 mM NaHCO3 containing protease inhibitors (cOmplete, Roche)), lysed in 2x SDS-sample buffer (2 µl per embryo) for 5 min at 95 °C and subjected to standard western blot analysis (12 µl per lane equals ~6 embryos) using the following antibodies: pAb-UL97 (kindly provided by D.M. Coen, Harvard Medical School, Boston, MA, USA), mAb-GFP (Roche), mAb-FLAG (Sigma-Aldrich), and mAb-α-tubulin (Abcam).

### pUL97 kinase using *in vitro* kinase assay

The viral kinase pUL97 was transiently expressed for two days in 293T cells by transfection of the plasmid constructs coding for pUL97-Flag (wild-type), pUL97(K355M)-Flag (catalytically inactive), pUL97-GFP (C-terminal fusion of the EGFP sequence) and pUL97(181–707)-Flag (N-terminal truncation of amino acids 1–180). Vector pcDNA3.1+ was used as a negative control. The kinase activity of pUL97 was determined *in vitro* after immunoprecipitation from transfected 293T cells as described^41^.

### Statistical analysis

Data are expressed as the mean ± SD of at least three independent experiments. Statistical significance of differences was evaluated by one-way ANOVA followed by Bonferroni’s post hoc test or by two-tailed t-test (IBM SPSS Statistics, version 21). p < 0.05 was considered statistically significant.

## Supplementary information

Supplementary Information
