# Supplementary material for: Human cytomegaloviral multifunctional protein kinase pUL97 impairs zebrafish embryonic development and increases mortality
Source: Sci Rep. 2019 May 10;9:7219. doi: 10.1038/s41598-019-43649-x (PMC6510723; doi:10.1038/s41598-019-43649-x)

Supplementary information for

Human cytomegaloviral multifunctional protein kinase pUL97 impairs zebrafish embryonic development and increases mortality

Salvador Cazorla-Vázquez, Mirjam Steingruber, Manfred Marschall, Felix B. Engel

Content:

1) Supplementary Figure Legends

2) Supplementary Figure 1

3) Supplementary Figure 2

4) Supplementary Figure 3

Supplementary Figure Legends

Supplementary Figure 1: Ectopic expression of pUL97 in zebrafish embryos. Single cell-staged embryos were injected with nuclease-free water (diluent) or plasmids (50 ng/µl) encoding eGFP and pUL97-eGFP. (**a**) Western blot analysis using total lysates of zebrafish embryos (~6 embryos per lane) probed with anti-pUL97 and anti-GFP antibodies.

Supplementary Figure 2: Global RNA expression of pUL97 in zebrafish embryos. Single cell-staged embryos were injected with nuclease-free water (diluent) or RNA (200 pg) encoding eGFP, pUL97-eGFP or pUL97(K355M)-eGFP. (**a**) Quantitative analysis of phenotype classes at 24 hpf. (**b**) Fluorescence images at 24 hpf indicating global expression of eGFP, pUL97-eGFP and pUL97(K355M)-eGFP. For each condition and independent experiment 100 to 128 embryos were analyzed (in total 1012 embryos in three independent experiments). Data are mean ± SD. Scale bars: 250 µm. ***: $p < 0.01$.

Supplementary Figure 3: Analysis of the catalytic activity of four different versions of the viral pUL97 kinase using in vitro kinase assay. The viral kinase pUL97 was transiently expressed for two days in 293T cells by transfection of the plasmid constructs coding for pUL97-Flag (wild-type), pUL97(K355M)-Flag (catalytically inactive), pUL97-GFP (C-terminal fusion of the EGFP sequence) and pUL97(181-707)-Flag (N-terminal truncation of amino acids 1-180). Vector pcDNA3.1+ was used as a negative control. (**a**) Total cell lysates were subjected to immunoprecipitation using mAb-Flag or mAb-GFP antibodies. The immunoprecipitates were analyzed for pUL97 autophosphorylation activity using the *in vitro* kinase assay. Reaction products were separated by SDS-PAGE and processed by western blot transfer and subsequent exposure to phospho-imager plates. As an input control, one-twentieth volume of each total cell lysate was directly subjected to SDS-PAGE and used for parallel western blot staining using the indicated antibodies.

Cazorla-Vázquez *et al.*, Figure S1

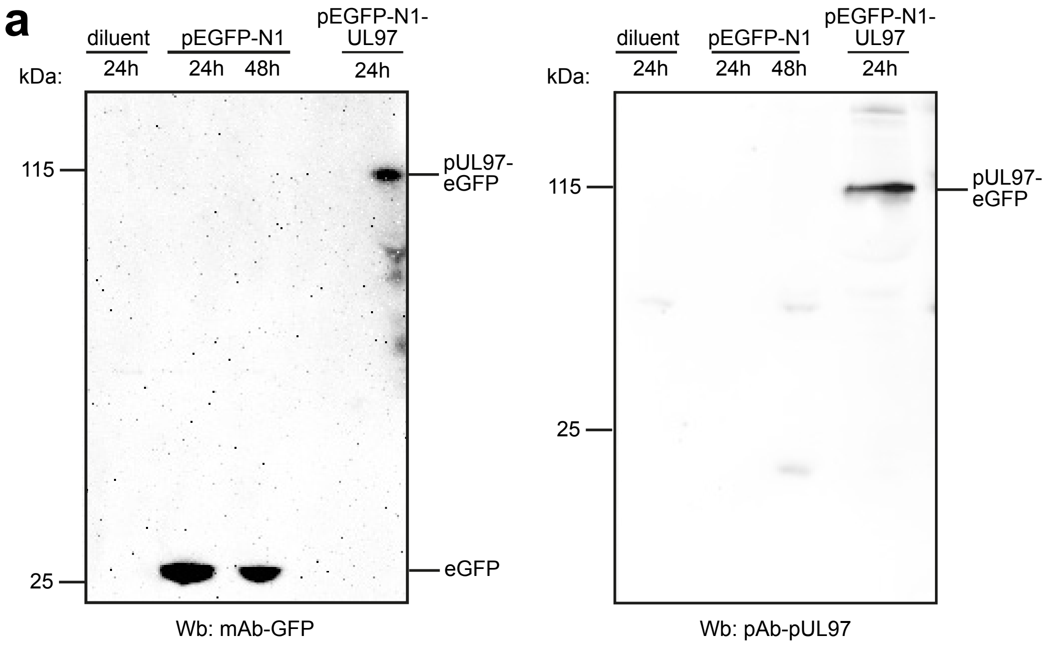

Cazorla-Vázquez *et al.*, Figure S2

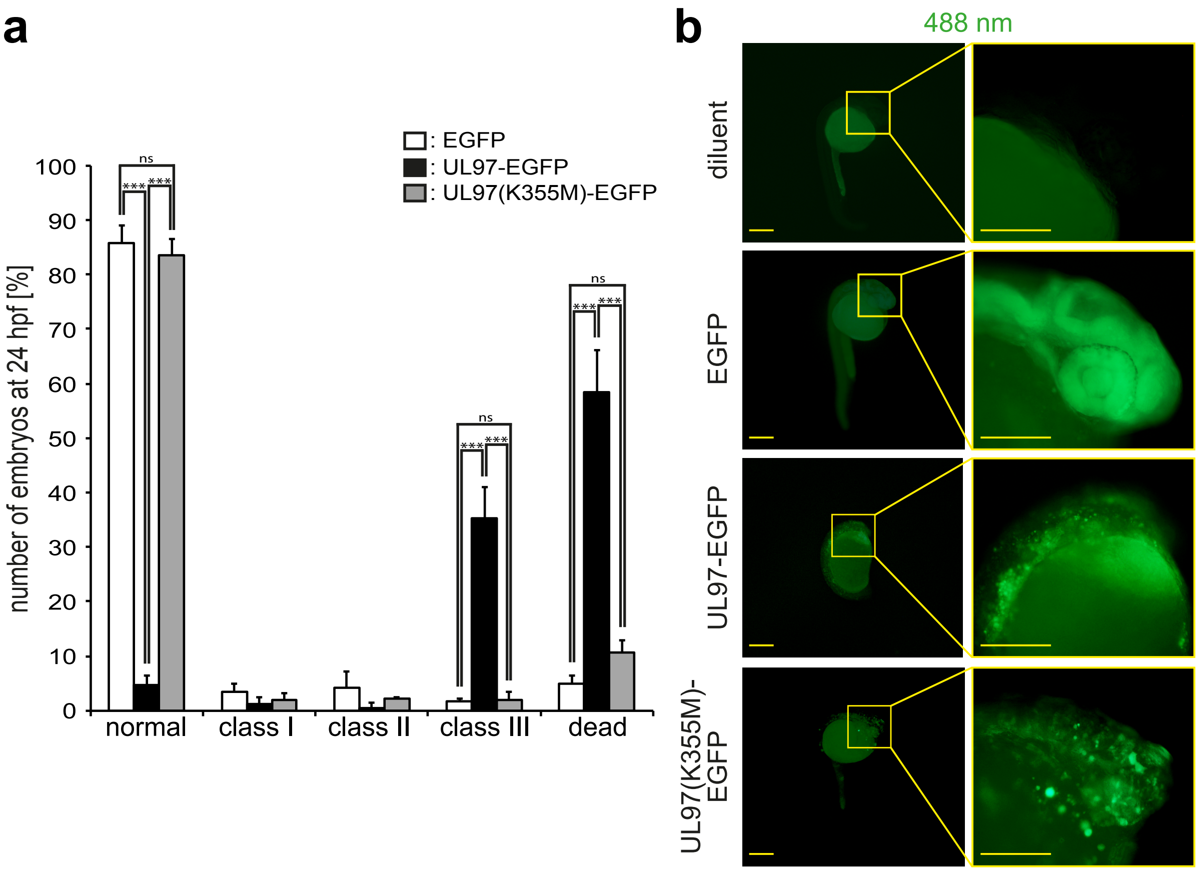

Cazorla-Vázquez *et al.*, Figure S3

a

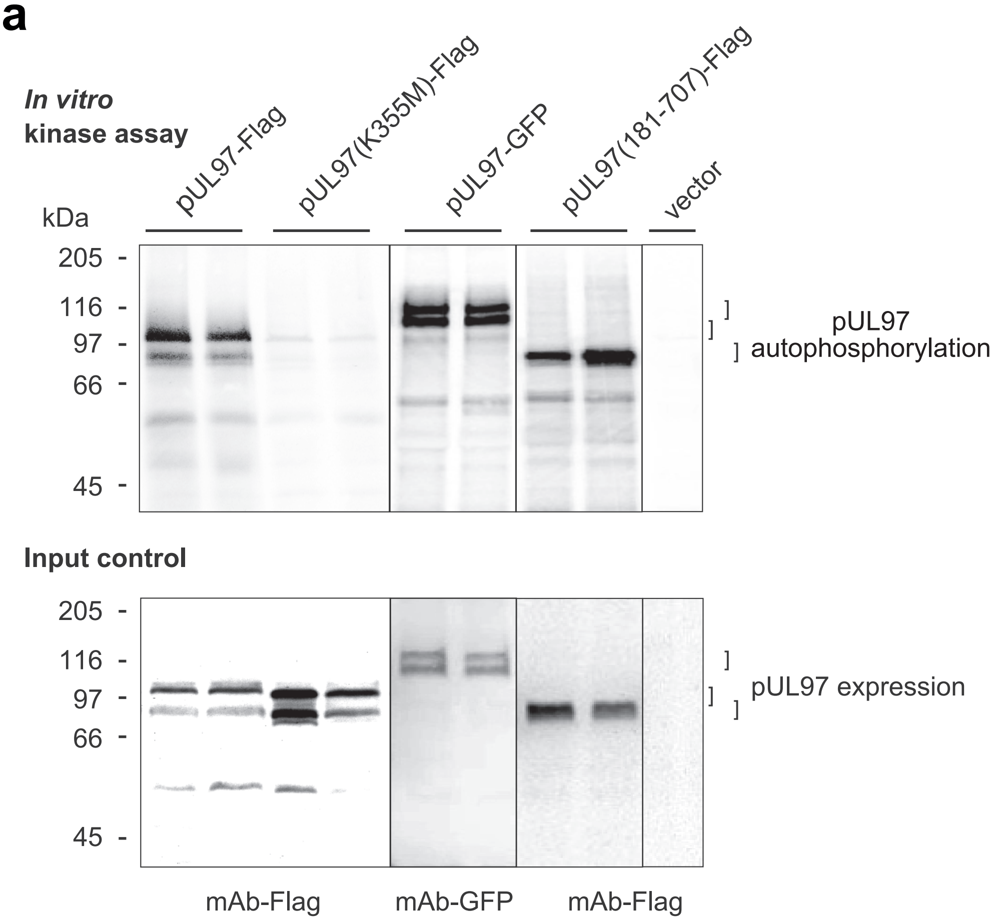

Supplement: Supplementary file 1 — Supplementary Information [file 41598_2019_43649_MOESM1_ESM.pdf]
